# Chronic Ketosis Provides Neuroprotection Through HIF- 1α-Mediated Control of the TXNIP/NLRP3 Axis by Regulating the Inflammatory and Apoptotic Response

**DOI:** 10.1007/s12035-025-04943-0

**Published:** 2025-04-24

**Authors:** Kehkashan Parveen, Mohd Salman, Golnoush Mirzahosseini, Arshi Parveen, Tauheed Ishrat, Michelle A. Puchowicz

**Affiliations:** 1 Department of Pediatrics, The University of Tennessee Health Science Center, TSRB, 71 S Manassas St, Suite 425, Memphis, TN 38163 USA; 2 Department of Anatomy and Neurobiology, The University of Tennessee Health Science Center, Memphis, TN USA; 3 Department of Anatomy and Neurobiology, College of Medicine, University of Tennessee Health Science Center, 875 Monroe Avenue, Wittenborg Bldg, Room- 231, Memphis, TN 38163 USA

**Keywords:** HIF- 1α, Ischemic stroke, Ketogenic diet, NLRP3 inflammasome

## Abstract

We and others have previously demonstrated that hypoxia-inducible factor alpha (HIF-1α) stabilization through diet-induced ketosis plays a vital role during brain ischemic injury. We have recently reported that ketosis-stabilized HIF-1α regulates the inflammatory response and contributes to neuroprotection in a rat stroke model. In the current investigation, we examined the downstream mechanism by which the ketogenic (KG) diet protects against brain damage after stroke in mice. Six- to seven-week-old male mice were fed the standard diet (SD) or the KG diet to mimic the metabolic state of chronic ketosis. After 4 weeks, mice were subjected to photothrombotic ischemic stroke. Behavior analysis was recorded at 24 h, 48 h, and 72 h post-stroke. After 72 h, mice were euthanized for infarction, brain edema, hemorrhage, and molecular analysis. Our results showed that the KG diet significantly alleviated infarction, brain edema, and hemorrhage; improved the neurobehavioral outcomes; and attenuated ischemic stroke-induced oxidative/nitrative stress and apoptotic markers at 72 h post-stroke. Further, the KG diet upregulated the HIF-1α and interleukin (IL)-10 expression and inhibited thioredoxin-interacting protein (TXNIP), NOD-like receptor family pyrin domain-containing 3 (NLRP3) inflammasome activation and pro-inflammatory cytokine expression compared to SD-fed mice after stroke. We showed that the KG diet did not show neuroprotection in the NLRP3 knockout mice after stroke. Our current study demonstrates that the KG diet exerts neuroprotective effects by inhibiting TXNIP-NLRP3 inflammasome, mainly dependent on heightening the upregulation of IL-10 via HIF-1α stabilization. Thus, the KG diet might be considered a new therapeutic strategy for ischemic patients.

**Supplementary Information:**

The online version contains supplementary material available at 10.1007/s12035-025-04943-0.

## Introduction

 Ischemic stroke is the most common cerebrovascular disorder [1]. It is the second major health issue that poses a threat to life, and its burden is rising quickly worldwide [2, 3]. Several immune cells migrate to the ischemic lesion after ischemic stroke and cause the release of a variety of neurotrophic factors, each of which has a positive or negative effect on brain tissue [4–6]. Secondary post-ischemia neuroinflammation causes additional damage and cell apoptosis [7]. The lack of effective therapeutic options is the primary obstacle in the fight against stroke. The only FDA-approved treatments for ischemic stroke are thrombolysis and endovascular thrombectomy; however, these treatments are only available to a small percentage of stroke patients because of their limited effectiveness and the specialized procedures required to perform them [8, 9]. Thus, the primary goal of stroke research is to develop cost-effective and viable treatments that alleviate brain dysfunction caused by ischemic stroke. This can only be done by understanding the underlying molecular events more deeply.

Previous reports have shown the contribution of inflammation in the pathogenesis of ischemic stroke, whereas NOD-like receptor family pyrin domain-containing 3 (NLRP3) inflammasome plays a crucial role [10–12]. The cytosolic pattern recognition receptor NLRP3 recruits the adapter protein apoptosis-associated speck-like (ASC) pro-caspase- 1 in response to several stroke-induced stimuli. This results in the production of caspase- 1 and the subsequent maturation and release of interleukin- 1β (IL- 1β) [13]. In acute stroke, the significance of pro-inflammatory and pro-apoptotic actions of IL- 1 β is well-supported [14, 15]. Additionally, irrespective of IL- 1β, the activated caspase- 1 results in pyroptotic cell death, which is known to cause a significant release of cytokines through intramembranous pores in glial cells [16]. According to recent studies, the suppression of NLRP3 inflammasome modulates ischemic insult and neurovascular consequences of experimental stroke [17–19]. Yet, because they mostly deal with non-specific neuroprotectants or genetic regulation, they fail to account for the therapeutic benefits. As a result, the need to create novel NLRP3 inhibitors with sufficient biocompatibility for clinical trials has been encouraged.

The primary feature of ischemic stroke is artery obstruction, which can cause blood flow disturbance or a drop in blood glucose levels, both of which increase the risk of brain damage. Ketone bodies, also known as beta-hydroxybutyrate and acetoacetate, or BHB and AcAc, are alternative energy substrates the brain uses well [20]. Treating drug-resistant pediatric epilepsy with the ketogenic (KG) diet is a well-known non-pharmacological approach that has shown promise in treating other neurological illnesses such as stroke and Alzheimer’s disease [21, 22]. We have consistently demonstrated that local intraventricular BHB infusions or the KG diet-induced ketosis correlate with neuroprotection after ischemic stroke. [23, 24]. Moreover, the KG diet can stop neuronal death brought on by hypoxia or glucose restriction [23]. Although the KG diet has been used in therapeutic settings for more than 70 years, the precise mechanism through which it gives neuroprotection is still unknown. Studies have linked ketosis to altering inflammatory and metabolic pathways [24–27]. Reports have shown that hypoxia-inducible factor- 1 alpha (HIF- 1α) accumulation elicits a neuroprotective response by modifying inflammatory pathways via modulation of cytokine regulation [28, 29]. Current research links the stabilization of HIF- 1α by ketosis as a potential neuroprotective trait in mice via the activation of the Jak1-Stat3 or Akt/Erk pathways by IL- 10 [30, 31]. Earlier, we investigated how the KG diet affected inflammatory reactions linked to stabilizing HIF1-α in the preconditioned ketotic rat brain. To identify putative neuroprotective pathways mediated by HIF1-α, we have focused on markers of pro- versus anti-inflammatory cytokines (TNF-α and IL- 6 vs IL- 10) in the cortical brain of ketotic rats [26, 32].

Based on these previous reports, the present study was designed to investigate the potential mechanisms associated with stabilizing HIF- 1α and IL- 10-mediated downregulating inflammation in the brains of preconditioned ketotic mice.

## Methods

### Animals and Experimental Groups

The experimental procedures were approved by the ARRIVE (Animal Research: Reporting In Vivo Experiments) guidelines and the Institutional Animal Care and Use Committee (IACUC) at the University of Tennessee Health Science Center in Memphis, TN, USA. Two major experiments were conducted. WT Male CB57BL/6 J mice (6–7 weeks old) were obtained from the Jackson Laboratory, Bar Harbor, ME, USA. For 4 weeks, the mice were fed either the KG (high fat, carbohydrate restriction) or standard lab-chow (STD) diets before the brain tissues were collected, as previously described [26]. The WT mice were randomized and divided into four groups: sham, pMCAO (mice with standard diet + photothrombotic middle cerebral artery occlusion), KG + pMCAO (mice with KG diet + photothrombotic middle cerebral artery occlusion), and KG (mice with KG diet only). The mice were kept in a standard environment with 45–50% humidity, 22–25 °C temperature, and a 12–12 light–dark cycle. They were also given unlimited access to food and drink. Diet protocols were followed, including the standard (STD; 27.5 fat%, 20.0 protein%, 52.6, given by the UTHSC animal facility) and ketogenic (KG; 89.5 fat%, 10.4 protein%, 0.1 CHO%; Research Diets, New Brunswick, NJ diet). In experiment 2, NLRP3 knockout mice were divided into two groups. They were fed either STD or KG diet. All mice were fasted overnight before receiving their diets to maintain blood glucose levels and start the ketosis process. A small blood sample from the tail was used to analyze weekly BHB concentrations using a keto-meter (Precision Xtra, Abbott, Alameda, CA).

### Photothrombotic Stroke Surgery

The photothrombotic stroke model was chosen for the current research because it has accuracy, repeatability, and consistency of the infarct volume and allows for visual confirmation of middle cerebral artery occlusion and no fatality rate (MCAO) [33]. The photothrombotic procedure was performed as we described, with minor alterations. After being given isoflurane anesthesia (3% for induction and 1.5% during the procedure), pMCAO and KG + pMCAO mice underwent photothrombotic surgery. To expose the artery, the head was shaved, and the left side of the scalp, between the ear and the eye, was cut. Through the retroorbital sinus, 15 mg/kg of rose bengal, a photothrombotic dye (RB, Sigma-Aldrich, Cat#330000), was injected. The middle cerebral artery was then exposed to a high-intensity photobeam for 4 min. Following surgery, the mice were placed in new cages in groups of 2 to 5 animals each, with free access to food and water. Until the study’s last day, researchers looked for any signs of discomfort or distress. As considered necessary, painkillers and fluid food supplements were given. An intraoperative rectal probe and feedback control pad were used to maintain the patient’s body temperature at 37∓ 0.5 °C. Mice were anesthetized using isoflurane and then transcardially infused with regular cold saline after 72 h of pMCAO. Body weight changes were recorded weekly and after induction of pMCAO at 0 h, 24 h, 48 h, and 72 h in all animals. Mice were then decapitated, and the brains were removed.

### CatWalk Test

Using the CatWalk XT automated analysis device (Noldus Information Technology, Wageningen, The Netherlands), gait analysis was carried out at 24 h, 48 h, and 72 h post-stroke to compute the designated parameters previously described [10]. Before performing the baseline assessment, the experimental animals were trained to traverse the walkway for 5 days in a row for 30 min. The system’s centerpiece is a promenade with a glass surface illuminated by green and red ceiling light sources. The CatWalk XT technology uses a camera underneath a glass walkway to take images of animal footprints and foot force profiles. When an experimental animal walks across the walkway’s glass plate, its paw prints are illuminated by the green light source. In contrast, the ceiling light contrasts the animal’s body contour. These events were captured by a fully automated camera mounted below the glass plate and then processed by the system’s software. A description of parameters is given in (Suppl. Table.S1). Pressure-related parameters, including mean intensity, which shows the complete paw’s mean intensity, were measured [34]. We collected three trials of each animal and mean ± SEM was used to calculate and graph the experimental data.

### Rotarod Test

The rotarod test was performed on experimental animals using rotarod equipment (Med Associates Inc., USA; model ENV- 577 M). In brief, the experimental animals were trained 3 days before the surgery at a speed of 30 rpm/min for 4 min, with an acceleration from 2 to 30 rpm/min within the same time frame. The rotarod was used to evaluate mice’s limb motor coordination and stability and the effect of the KG diet following ischemic stroke. The rotarod test was carried out at different time points (24 h, 48 h, and 72 h), and the latency to fall was recorded for two maximum testing times out of 3 when the mice fell off from the rotating rod. Results were expressed as the time (sec) spent on the rotarod.

### Infarct Size and Brain Edema

A blinded researcher measured infarction and cerebral edema. The brain was separated following cardiac perfusion with ice-cold normal saline. Six 1-mm-thick coronal slices from each brain were then stained for 15 min at 37 °C using 1% TTC solution (2,3,5-triphenyl tetrazolium chloride-Sigma-Aldrich, St. Louis, MO) and scanned. As we previously reported, the ImageJ software was used to measure the infarct size and both hemisphere regions blindly [35]. The area difference between the contralateral and ipsilateral hemispheres was used to quantify hemispheric edema.

### Hemoglobin (Hb) Excess and Hemorrhagic Transformation

A colorimetric hemoglobin detection technique (QuantiChrom Hemoglobin technique Kit, BioAssay Systems, Haywood, CA) was used to assess hemorrhagic transformation following microcarcinomal infarction of the brain and cerebrovascular disruption [36]. In a 10% glycerol Tris-buffer saline solution with 0.5% Tween 20, ipsilateral TTC-brain slices were homogenized. Subsequently, brain samples were subjected to a standard microplate reader (Synergy HT, BioTek equipment) reading at 562 nm for colored hemoglobin. Following the manufacturer’s directions, the hemoglobin concentration was determined and expressed in μg/dL using standard samples.

### Western Blot Analysis

The cortical brain’s nuclear protein extracts were immunoblotted with anti-Hif1α normalized to TATA-box binding protein (TBP). Meanwhile, whole cell RIPA® lysates were immunoblotted for other proteins. Briefly, 50 μg protein was loaded into each lane, separated, and then transferred to PVDF membranes. The membranes were blocked with non-fat dry milk (5% in TBST) for 2 h at room temperature to avoid the nonspecific binding and probed with primary antibodies against NLRP3, cleaved caspase- 1, ASC (1:1000; AG- 20B- 0014; AG- 20B- 0042; AG- 25B- 0006 Adipogen Life Sciences), TXNIP (1:1000; NBP1–54578SS; Novus Biologicals), Cl.PARP, (BD Bioscience Pharmingen, San Diego, CA), IL- 6, IL- 10, IL- 1β, TNF-α, caspase- 3 (1:1000; 12,912; 12,163; 12,242; 11,948; 9664; Cell Signaling Technology), TBP and β-actin (1:1000, T1827; A5316; Sigma) at 4 °C overnight. The next day, following twice TBST washes for 10 min, the membranes were incubated with horseradish peroxidase (HRP)-conjugated anti-mouse IgG antibody and anti-rabbit antibody (1:10,000; A9044; A9169; Sigma, USA) for 1 h at room temperature, as previously described [37]. The bands were visualized using an enhanced chemiluminescent substrate solution (Thermo Fisher Scientific). The optical density of samples was analyzed using the ImageJ software and normalized to the loading controls.

### Slot Blot

By using slot blot analysis, nitrotyrosine (NT), immunoreactivity was assessed as a commonly used indication of lipid peroxidation, 4-hydorxynonenal (4-HNE), and superoxide-dependent peroxynitrite production [38]. A slot blot microfiltration device was used to immobilize 20 μg of peri-infarct (penumbral) sample homogenates onto a nitrocellulose membrane. The membranes were then blocked with 5% nonfat milk and treated with either an anti-nitrotyrosine or anti- 4 hydroxynonenal antibody (1:1000; SMC- 511; Stress Marq Biosciences; 05–233; Millipore). Next, goat anti-mouse IgG labeled with peroxidase was added, and the membranes were detected using a Pierce Super Signal Kit. Using the densitometry ImageJ software, the optical density of several samples was measured.

### Statistical Analysis

Results were presented as mean ± SEM. Tukey’s post-hoc test assessed the differences between the experimental groups after the Student’s *t*-test or ANOVA. All statistical analyses were performed using GraphPad Prism 9.0 software (San Diego, CA, USA). Significance was defined by a *p* < 0.05.

## Results

### KG Diet Modulated Metabolic Panel in the pMCAO Group

Stroke led to body weight loss in pMCAO group animals but was insignificant (Supplementary material; Table-S2). After KG supplementation, body weight loss was significantly reserved following pMCAO at 72 h. We did not see any significant changes in body weight at earlier time points between the pMCAO and KG-supplemented pMCAO animals. Blood ketone levels in KG + pMCAO and KG group mice spiked in the first week of diet to 3.9 mM compared to sham and pMCAO mice and remained stabilized throughout the experiment to an average of 2.4 mM (Supplementary material; Figure-S1 A). Interestingly, the KG diet did not change fasting blood glucose levels (data not shown). At the same time, blood glucose concentrations were significantly lower in KG + pMCAO compared to the pMCAO group (Supplementary material; Fig-S1B).

### KG Diet Improved Sensory-Motor Performance in the pMCAO Group

**Fig. 1:**
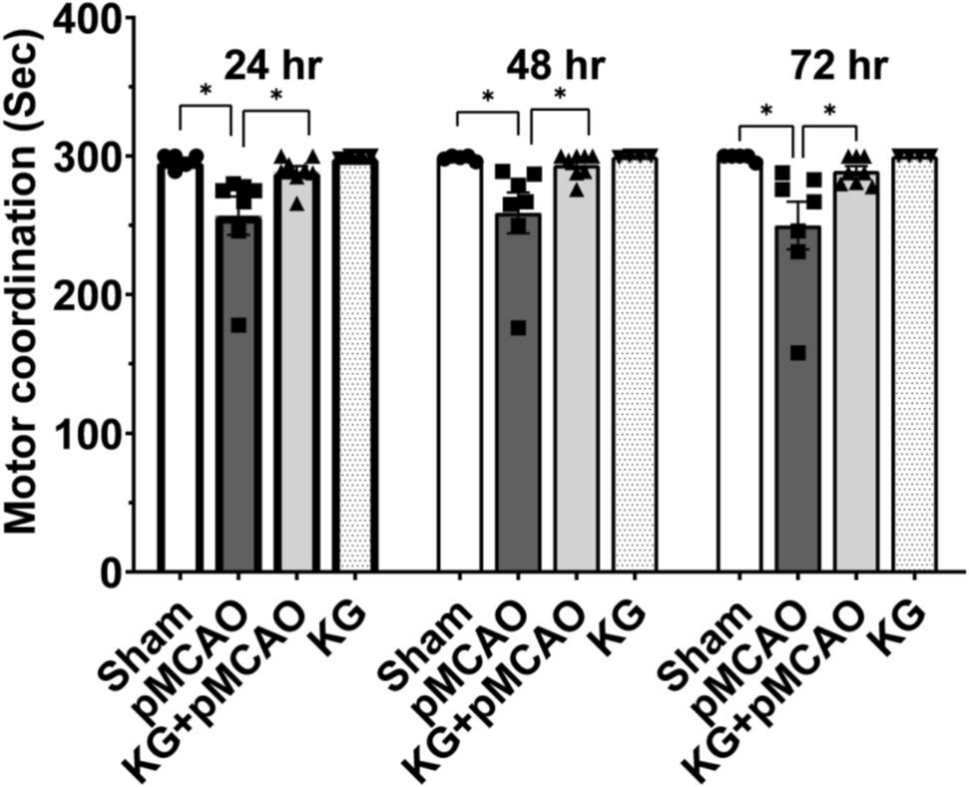
Effects of KG diet on the rotarod in pMCAO mice. pMCAO mice had significantly lower motor coordination than animals in the sham group. KG diet significantly improved rotometric performance compared with the pMCAO mice at different time intervals. *p < 0.05 compared sham vs. pMCAO group OR pMCAO vs. KG + pMCAO group. (*n* = 6–9); data are presented as mean ± SEM

**Fig. 2:**
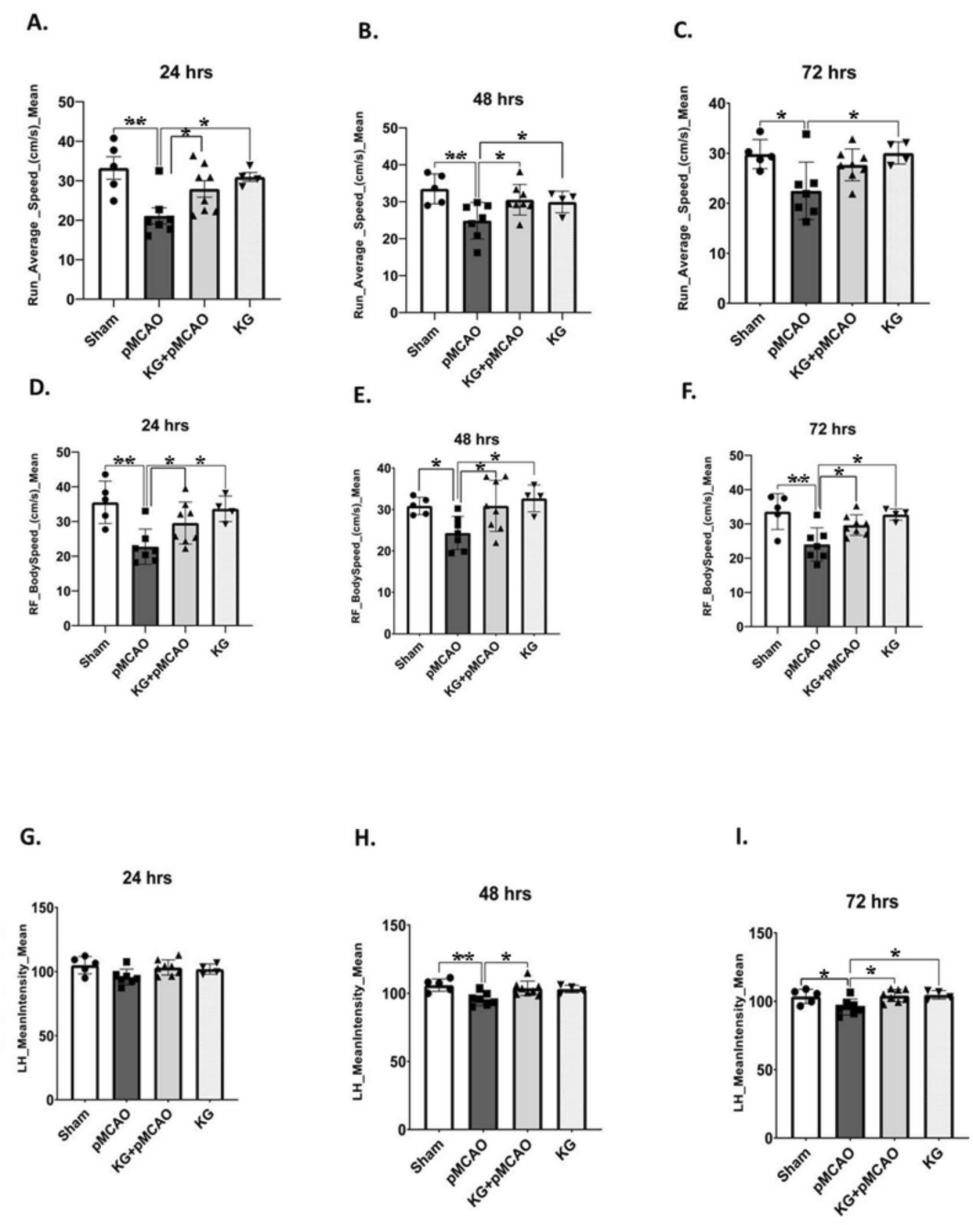

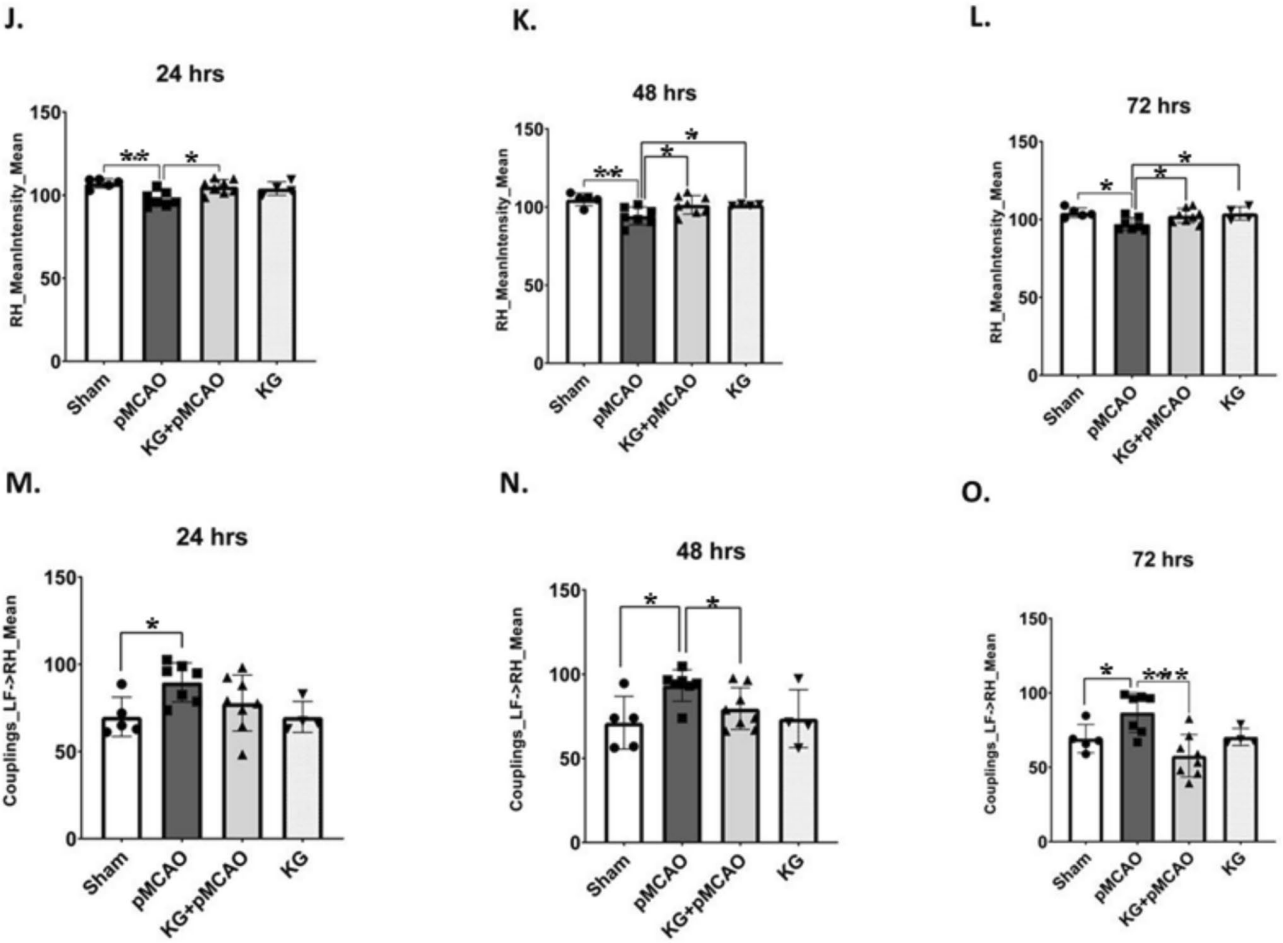
Effect of KG diet on the catwalk in pMCAO mice at 24 h, 48 h, and 72 h following photothrombotic stroke. **A**, **B**, and **C** A significant increase in run average speed in KG + pMCAO was observed. **D**, **E**, and **F** A significant increase in body speed in KG + pMCAO was seen. **G**, **H**, and **I** A significant increase in mean intensity for LH in KG + pMCAO was seen compared to pMCAO mic. **J**, **K**, and **L** A substantial increase in mean intensity for RH in KG + pMCAO was seen compared to pMCAO mice. **M**, **N**, and **O** Coupling LF − > RH markedly reduced in KG + pMCAO mice compared to pMCAO mice at different time points. Values are expressed as mean ± SEM (*n* = 6–9), *p < 0.05, **p < 0.01, and ***p < 0.001 compared sham vs. pMCAO group OR pMCAO vs. KG + pMCAO group

Motor coordination was evaluated by the maximum time spent on a rotating rod. pMCAO group animals showed motor impairment compared 

to the sham (Fig. 1). However, KG preconditioning in the KG + pMCAO group significantly improved motor coordination (*p* < 0.05). Our catwalk findings showed a marked difference in speed-dependent parameters, including average run speed and body speed in pMCAO and KG + pMCAO mice. pMCAO mice (*p* < 0.01) significantly showed less run average rate than sham following injury. Meanwhile, KG diet–fed mice showed remarkably increased run average speed at different time points post-pMCAO (Fig. 2A, B, and C). Furthermore, pMCAO mice markedly walked slower than sham, whereas KG + PMCAO significantly (*p* < 0.05) recovered and restored their speed in the right front paw (RF) (Fig. 2D, E, and F). This result exhibits that pMCAO mice were poorly performed due to the brain injury, and it took the system more than 2 s to record the run. The mean intensity was significantly higher in KG + pMCAO compared to pMCAO mice for the left hind paw (LH) and right hind paw (RH) (Fig. 2G, H, I, J, K, and L) at each time point post-injury. This result suggests that pMCAO mice avoided putting high pressure on their paws while running in the walkway. We also detected that there is a slight increase in coupling in pMCAO mice in left front paw to right hind paw (LF- > RH) in comparison to sham, and KG + pMCAO mice markedly reduced the coupling post-injury at different time points (Fig. 2M, N, and O).

### KG Diet Reduced Infarction, Brain Edema, and Hemorrhage in the pMCAO Group

**Fig. 3:**
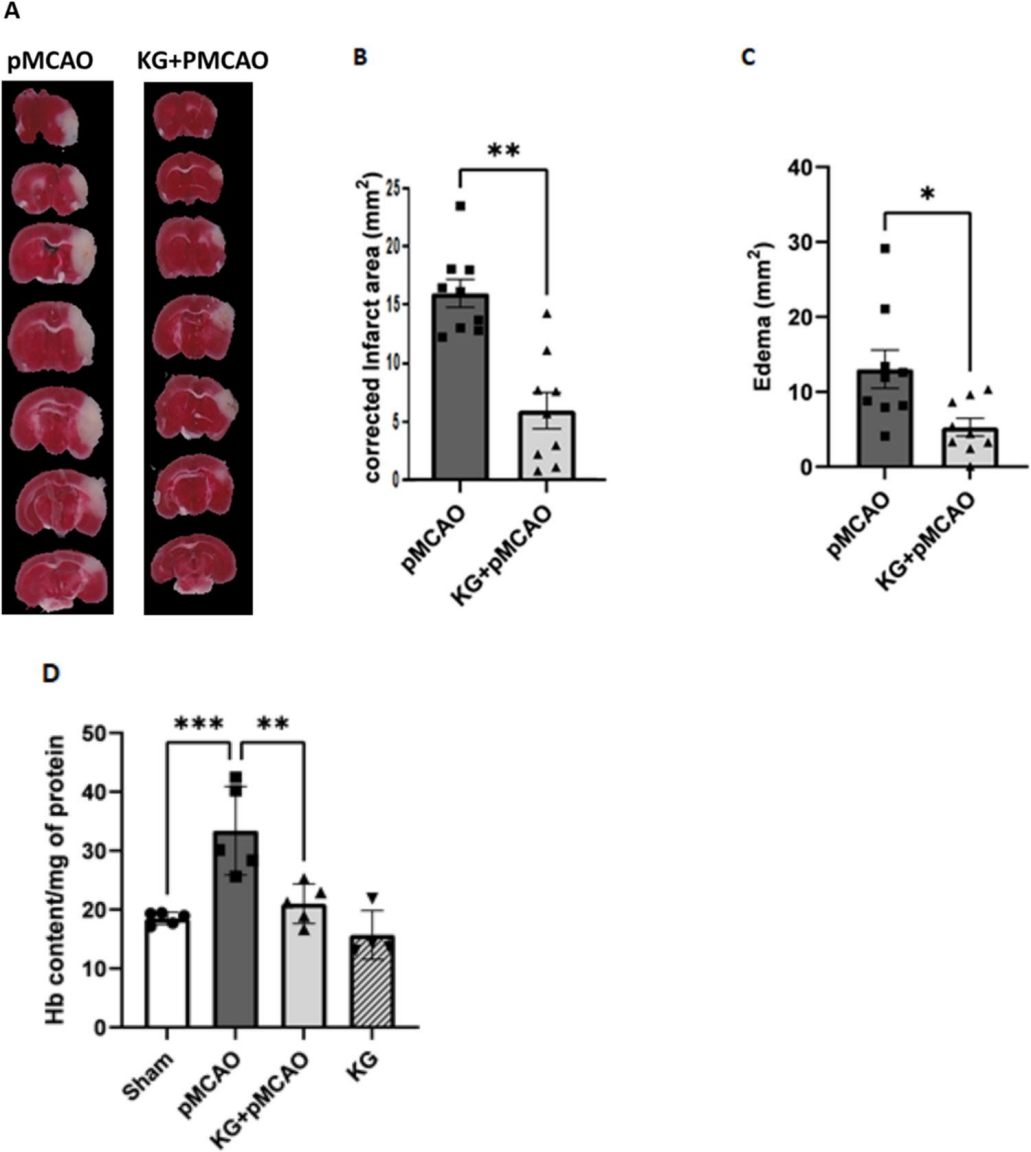
The KG + pMCAO group showed less infarct size, cerebral edema, and brain hemorrhage compared to the pMCAO mice after 72 h following photothrombotic stroke. **A** TTC sections of brain samples in pMCAO and KG + pMCAO group. **B** KG + pMCAO showed a remarkable reduction in infarct size compared to the pMCAO. **C** KG + pMCAO represented a significant decrease in ipsilateral edema post-injury. **D** KG + pMCAO significantly reduced cerebral hemorrhage compared to the pMCAO. Values are expressed as mean ± SEM (*n* = 6–9), *p < 0.05, **p < 0.01, and ***p < 0.001 compared sham vs. pMCAO group OR pMCAO vs. KG + pMCAO group

Coronal brain slices stained with TTC revealed white infarcted areas following 72 h of pMCAO, as illustrated in Fig. 3A. The pMCAO group’s dead brain tissue and ipsilateral edema were correlated with the stroke animals’ visible infarction regions, which suggested brain injury and neuronal cell death. Nevertheless, supplementation with the KG diet effectively reduces infarct size and edema, improving functional outcomes and providing neuroprotective effects against ischemic stroke, as shown in Fig. 3B and C. To examine the effects of the KG diet on hemorrhage, brain tissue Hb content was estimated as an index for the incidence of intracerebral hemorrhage in perfused brains at 72 h after pMCAO (Fig. 3D). The Hb content was significantly (*p* < 0.01) reduced in the KG + pMCAO group compared to pMCAO.

### KG Diet Modulated HIF1α-Mediated Inflammatory Response in the pMCAO Group

**Fig. 4:**
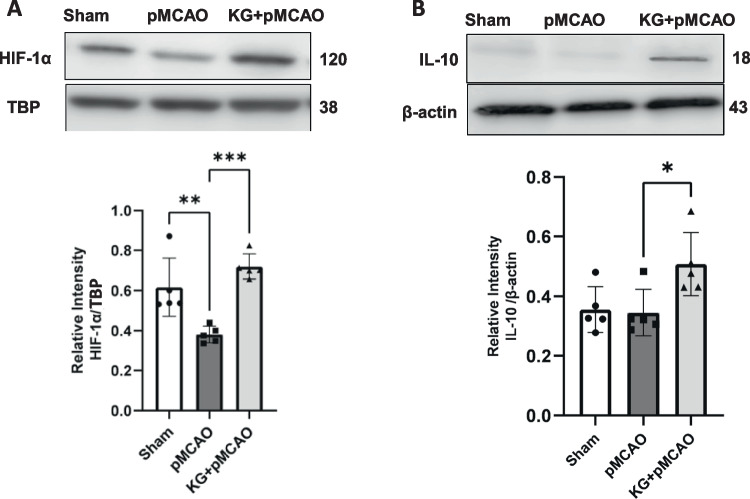
The involvement of HIF- 1α modulation with IL- 10 in KG-supplemented animals following photothrombotic stroke. **A** pMCAO group showed significantly reduced HIF- 1α level, which was upregulated remarkably in the KG diet supplementation group. **B** There was little change in protein expression of IL- 10 in pMCAO mice after 72 h of stroke induction, while it was upregulated significantly in KG-fed mice. Values are expressed as mean ± SEM (*n* = 5–7), *p < 0.05, **p < 0.01, and ***p < 0.001 compared sham vs. pMCAO group OR pMCAO vs. KG + pMCAO group

**Fig. 5:**
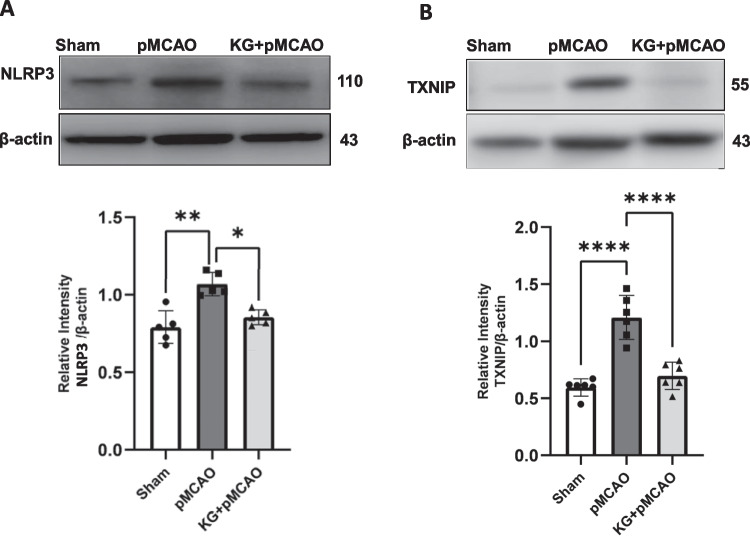
KG diet supplementation attenuates NLRP3/TXNIP inflammasome activation after photothrombotic stroke. **A** pMCAO significantly activated NLRP3 in ipsilateral sections modulated in KG diet–supplemented mice in the KG + pMCAO group. **B** TXNIP protein level was significantly higher in the pMCAO group and lower in the KG + pMCAO group. Values are expressed as mean ± SEM (*n* = 5–7), *p < 0.05, **p < 0.01, and ***p < 0.001, ****p < 0.0001 compared sham vs. pMCAO group OR pMCAO vs. KG + pMCAO group

**Fig. 6:**
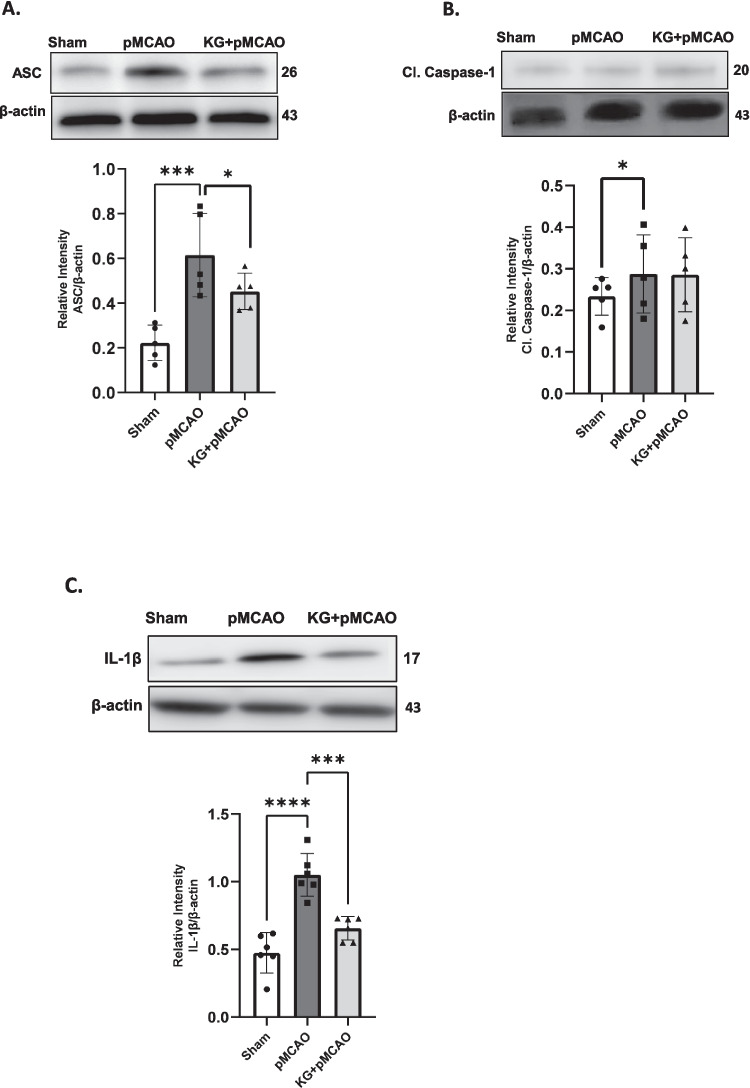
Further involvement of NLRP3 inflammasome assembly indicated that pMCAO mice significantly showed upregulation of ASC (**A**), caspase- 1 (**B**), and IL- 1β (**C**), which were downregulated remarkably in KG + pMCAO mice. Values are expressed as mean ± SEM (**n** = 5–7), *p < 0.05, **p < 0.01, ***p < 0.001, and ****p < 0.0001 compared sham vs. pMCAO group OR pMCAO vs. KG + pMCAO group

**Fig. 7:**
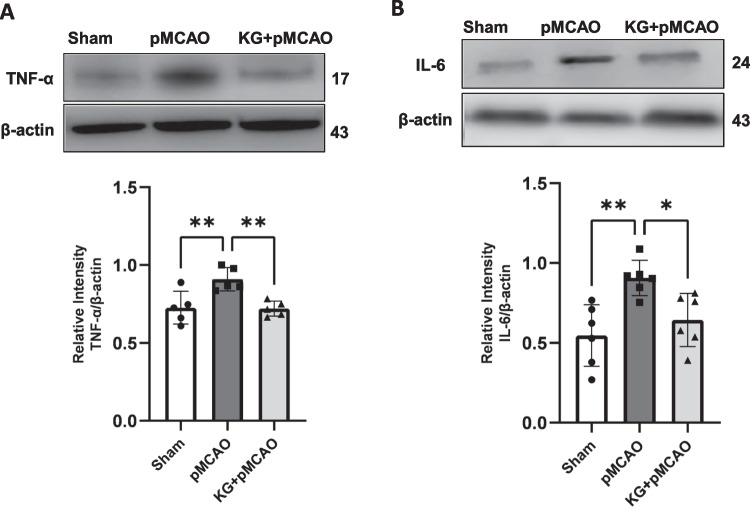
The effect of KG diet on pro-inflammatory cytokines after photothrombotic stroke. Expression of pro-inflammatory cytokines TNF-α and IL- 6 (**A** and **B**) was significantly higher in pMCAO mice and downregulated in KG + pMCAO mice. Values are expressed as mean ± SEM (*n* = 5–7), *p < 0.05, **p < 0.01, ***p < 0.001, and ****p < 0.0001 compared sham vs. pMCAO group OR pMCAO vs. KG + pMCAO group

Immunoblotting results (Figs. 4A and B) show significantly (*p* < 0.001) higher protein levels of HIF1α in the cortical brain of KG + pMCAO mice compared to the pMCAO group. In contrast, its level was diminished significantly (*p* < 0.01) in pMCAO compared to the sham group. IL- 10 levels were significantly higher in the cortical brain of KG preconditioning mice (KG + pMCAO) compared to stroke-induced mice maintained on an STD diet (pMCAO). We used western blotting analyses to detect the expression of NLRP3 inflammasome-associated proteins in the ipsilateral brain tissue. These proteins include NLRP3, TXNIP, cleaved-caspase- 1, apoptosis-associated speck-like protein (ASC), and IL- 1β. It is thought that NLRP3 signaling proteins contribute to the development of ischemic brain injury (Figs. 5A and B and 6A-C). Stroke triggered the NLRP3 inflammasome activation by upregulating NLRP3 inflammasome-associated proteins, as shown in Fig. 6A–C. However, these proteins were significantly downregulated in KG diet–fed animals. Although, we found a non-significant downregulation of cleaved caspase 1 (Fig. 6B). The effect of the KG diet on TNF-α and IL- 6, both pleiotropic cytokines that rapidly upregulate in the brain after injury, was also examined. Our immunoblotting showed that TNF-α and IL- 6 were highly expressed in the pMCAO group compared to the sham (Figs. 7A and B). In the KG diet preconditioning group (KG + pMCAO), the TNF-α and IL- 6 levels were significantly (*p* < 0.01; *p* < 0.05 respectively) lower when compared to the pMCAO group. Our results showed that the KG diet fed reduced inflammation by inhibiting the activation of NLRP3 inflammasomes against ischemic stroke.

### KG Diet Attenuates the Activation of Caspase- 3 and PARP in the pMCAO Group

**Fig. 8:**
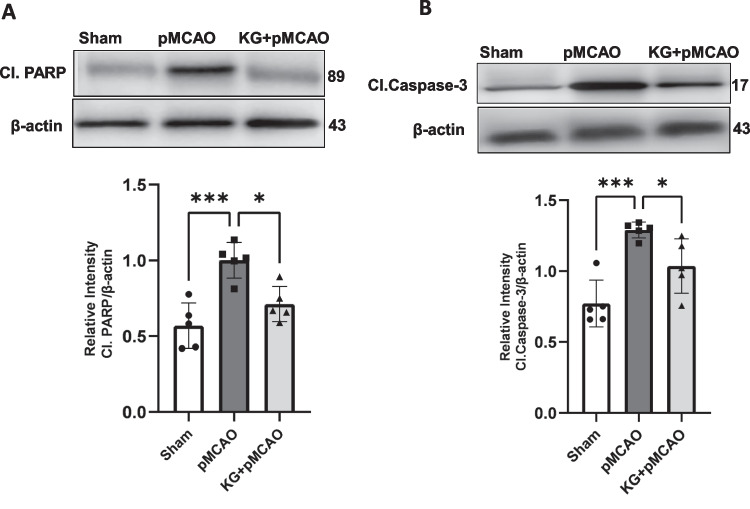
The contribution of KG diet in cell survival after photothrombotic stroke. **A** Increased expression of Cl.PARP was found in PMCAO mice markedly downregulated in KG + PMCAO mice 72 h post-stroke. **B** Stroke surgery markedly increased the expression of Cl. Caspase- 3 plays a role in apoptosis as pro-apoptotic. It was significantly reduced in KG + pMCAO mice following stroke surgery. Values are expressed as mean ± SEM (*n* = 5–7), *p < 0.05, **p < 0.01, ***p < 0.001, and ****p < 0.0001 compared sham vs. pMCAO group OR pMCAO vs. KG + pMCAO group

To examine the effect of the KG diet on neural apoptotic pathways, we next estimated the activation of the pro-apoptotic PARP and caspase- 3 at 72 h after pMCAO (Figs. 8A and B). In the ischemic condition, the activation of PARP following DNA damage might contribute to caspase- 3 activation. The expression of cleaved PARP and cleaved caspase- 3 were significantly (*p* < 0.001) increased after pMCAO compared to shams. Supplementation of the KG diet significantly (*p* < 0.05) reduced the activation of caspase- 3 expression, parallel with a marginal decrease in the activation of PARP in the KG + pMCAO group.

### KG Diet Ameliorated the Oxidative Damage in the pMCAO Group

**Fig. 9:**
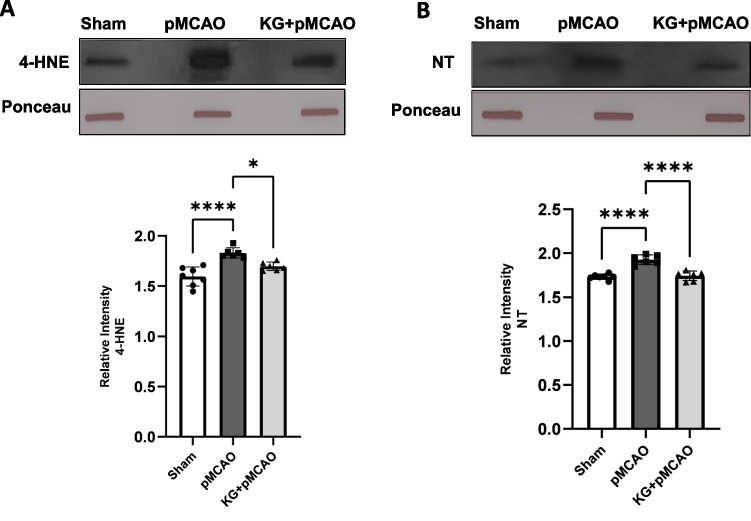
Effect of KG diet on the oxidative or nitrosative stress markers, nitrotyrosine (NT), and 4-hydroxynonenal (4-HNE). The level of NT and 4-HNE was significantly higher in pMCAO mice. A remarkably reduced level of these markers was observed in the KG + pMCAO mice. Values are expressed as mean ± SEM (*n* = 5–7), *p < 0.05, **p < 0.01, ***p < 0.001, and ****p < 0.0001 compared sham vs. pMCAO group OR pMCAO vs. KG + pMCAO group

Slot blotting results showed significantly (*p* < 0.0001) higher expression of the nitrotyrosine and 4-HNE in the pMCAO compared to the sham-operated group, whereas the KG diet showed a stronger inhibitory effect of both proteins compared with the pMCAO group (Fig. 9A and B). This might be explained by the KG diet, which could have antioxidant effects and reduced oxidative damage following ischemic stroke.

### Effect of Genetic Manipulation of NLRP3 on Metabolic Panel, Cerebral Infarction, Edema, and Sensory-Motor Performance Following pMCAO

NLRP3 knock-out mice showed a significant restoration of body weight in KG group animals (Suppl. material Fig. 2SA). After KG supplementation, body weight was significantly reserved following pMCAO at 72 h. We also observed significant changes in body weight before pMCAO in KG-supplemented animals compared to the STD group. Blood ketone level was non-significant in KG group mice compared to STD after 72 h (Suppl. Material Fig. 2SC). Interestingly, the KG diet did not change fasting blood glucose levels (data not shown) before PMCAO in KG group animals. At the same time, blood glucose concentration was significantly lower in the KG group compared to the STD group after 72 h (Suppl. material Fig. 2SB).

To confirm the role of NLRP3 in ischemic stroke in our findings, we examined whether the beneficial effect of KG diet was mediated via NLRP3 inflammasome using genetic knocking down of NLRP3 after ischemic stroke. According to our TTC sectioning results (Suppl. material Fig. 3SA), NLRP3 knockout mice exhibited non-significant cerebral infarct size and edema in either STD or KG group after photothrombotic stroke (Suppl. material Fig. 3SB). Our data also showed non-remarkable changes in cerebral edema in the KG group compared to 72 h after stroke (Suppl. material Fig. 3SC). Our catwalk findings represented that there was no marked difference in several speed-dependent, pressure, and coupling parameters, including average run speed, body speed, mean intensity, and coupling in STD or KG group animals (Suppl. material Fig. 3SD, E, F, G and H). Our data indicate that NLRP3 knockout mice not only had less ischemic infarct but also maintained better sensory-motor performance. Further, NLRP3 KO mice showed a decrease in infarct area compared to WT mice at 72 h after pMCAO (Suppl. material Fig. 3SI.).

## Discussion

An early occurrence in an ischemic stroke is inflammation-mediated cell dysfunction, which is a complex consequence of the emergence of neurological impairments [39]. Moreover, hypoxia and the formation of hypoxic signaling intermediates are known to accompany inflammatory conditions, which in turn initiate inflammatory responses by activating cytokines and inflammatory cells [40]. The current study’s findings demonstrated that hypoxia–ischemia insult activated the NLRP3 inflammasome in the photothrombotic stroke model in mice, resulting in brain damage. Preconditioning with the KG diet suppressed NLRP3 inflammasome activation through HIF- 1α-mediated downregulation of pro-inflammatory cytokines via IL- 10. We also found that these effects were associated with restoring neurobehavioural outcomes and improving brain injury in NLRP3 knockout mice.

The KG diet is a high-fat, extremely low-carbohydrate diet that causes the liver to produce more ketone bodies due to increased fat beta-oxidation. Ketone bodies (BHB, AcAc) are well used by the brain as energy substrates, particularly when there is a shortage of glucose, like when someone is chronically fasting or eating a restricted diet. Increased blood levels of ketone bodies resulted from ketosis, an alternative energy source to glucose and one that the brain is known to utilize efficiently [20]. When glucose metabolism is disrupted, such as when ischemia–reperfusion damage causes oxidative stress, ketones are advantageous substrates [41]. The KG diet is a well-known non-pharmacological strategy for treating drug-resistant epilepsy in children, and it has demonstrated potential in treating other neurological conditions such as Alzheimer’s and stroke [21].

We and others have consistently reported that the ketogenic state induced by the KG diet or calorie restriction or by infusions of BHB provides neuroprotection following cerebral ischemia in an experimental stroke model [26, 32, 42–45]. The current study further shows that a 4-week KG diet improved the mice’s brain ischemia tolerance to MCAO, as evidenced by improvements in the volume, edema, and Hb content of infarcts. In several mouse models of ischemic stroke, it is well-known that ischemic brain injury results in brain tissue destruction and neurobehavioral abnormalities [46]. In the present study, we demonstrated that photothrombotic stroke–induced neurobehavioral deficits and body weight loss. KG diet pre-supplementation improved the behavioral outcomes and body weight following photothrombotic stroke. Our findings are consistent with as previously reported [47]. In addition, our results showed that the genetic deletion of NLRP3 provides protection against ischemic brain damage. Moreover, in the present study, NLRP3 deficiency significantly reduced the infarct size and improved neurobehaviour outcomes following pMCAO.

The mechanism through which diet-induced ketosis confers neuroprotection remains unclear despite its many emerging pre-clinical or clinical investigations [26]. Data from previous studies have demonstrated that the KG diet exerts a neuroprotective effect by alleviating inflammatory pathways [24–27]. Puchowicz et al. (2008) reported that the HIF- 1α level was elevated after the KG diet because increased succinate inhibited the prolyl hydroxylase, an enzyme responsible for the degradation of HIF. Previously, studies have suggested that HIF- 1α can function as a transcriptional activator of anti-inflammatory cytokines, including IL- 10, by binding to hypoxia-responsive elements (HREs) in their promoter regions [30, 31]. In our study, we also found the upregulation of HIF- 1α and enhanced expression of IL- 10 in mice pre-conditioning with KG diet. Recent studies combine ketosis-mediated stabilization of HIF- 1α as a potential neuroprotective phenotype in mice via IL- 10-mediated activation of Jak1-Stat3 and Akt/Erk pathways [30, 31]. Further, a recent study demonstrated that HIF- 1α mediates NLRP3 inflammasome-dependent cell death following ischemic stroke [48]. We also hypothesize that other inflammatory responses, especially the NLRP3 inflammasome, may be crucial in post-ischemic damage. Nevertheless, the effects of KG on the NLRP3 inflammasome in brain damage through HIF- 1α remain unclear.

The inflammatory responses to brain damage in the etiology of neurodegenerative disease and stroke have been implicated to be mediated by the NLRP3 inflammasome and its activation/related products, TXNIP, ASC specks, caspase- 1, and IL- 1β [19, 49]. Although NLRP3 deficiency decreased brain injury in and in an animal model of stroke, mounting evidence suggests that the levels of NLRP3-inflammasome and IL- 1 were elevated in the brain injury. As an indirect inhibitor of the NLRP3 inflammasome, resveratrol has been shown to have protective benefits against ischemic stroke injury in earlier studies [19, 50]. This study further explored the possibility of a connection between the reduction of the NLRP3-mediated inflammatory response and the neuroprotective benefits of KG diet preconditioning. Our findings showed increased TXNIP/NLRP3 inflammasome activation after pMCAO mice exhibited markedly elevated ASC and caspase- 1 activity. By downregulating the NLRP3, TXNIP, ASC, and Cl-caspase- 1 expression, the KG diet significantly decreased the activation of the NLRP3-inflammasome. According to Luheshi et al. (2011), early production of IL- 1β in areas of local neuronal injury underlines it as the major form of IL- 1, adding to inflammation early after ischemia [51]. Such a connection between NLRP3 inflammasome and cytokine release in the acute phase may help explain why the KG diet had such striking effects in our investigations. Earlier studies demonstrated that inhibition of the NLRP3 inflammasome is the primary reason for diminishing TXNIP and stroke-induced injury [11, 17, 52]. Consistent with earlier reports, our hypothesis supports that reduced NLRP3 inflammasome activation is coincident with neuroprotective modulations. Furthermore, we found a significant increase in TNF-α and IL- 6 in pMCAO stroke mice, which was significantly suppressed in KG diet pre-treated mice following PT stoke consistent with the previous studies [19]. The inverse relationship we observe with HIF1α and IL- 6 levels is similar to those previously reported. They reported that HIF1α protein levels were higher under sustained hypoxia with a significant reduction in both mRNA and protein levels of IL- 6 [15]. In the present pMCAO model, we could track a remarkable caspase- 1/IL- 1β repression followed by slightly diminished PARP and caspase- 3 cleavage, addressing confined inflammation and degeneration in KG diet–supplemented mice.

Inflammatory pathways that are mediated by IL- 10 have been thoroughly researched for their significance in the development of tailored strategies intended to regulate harmful inflammation in the brain [53]. IL- 10 acts as a negative regulator of the NLRP3 inflammasome, suppressing its activation and subsequent production of pro-inflammatory cytokines like IL- 1 beta, effectively limiting neuroinflammation. Prior studies have reported that IL- 10 inhibits NLRP3 inflammasome activation via Jak1-Stat3 signaling, reducing the expression of key inflammasome components such as NLRP3, ASC, and caspase- 1 [54, 55]. Furthermore, studies in ischemic and inflammatory models indicate that IL- 10 deficiency results in heightened inflammasome activation and increased IL- 1β production [53, 56]. Additionally, IL- 10 regulates the immune system by inhibiting the production of pro-inflammatory cytokines like IL- 6 and TNF-α through attenuation of the NLRP3 inflammasome [54], which is consistent with our findings.

Cerebral ischemia sets off a series of intricate biochemical and cellular reactions that lead to an overproduction of reactive species and oxidative damage, which is a key factor in stroke pathophysiology [35, 57]. It has been shown that excessive ROS/RNS generation during an acute ischemic stroke can overwhelm the body’s antioxidant defenses, harm macromolecules, including lipids, proteins, and nucleic acids, and ultimately result in neuronal damage [58]. Increased levels of 4-HNE, an index of lipid peroxidation, and nitrotyrosine, a nitrosative stress biomarker, were also among the indicators. Thus, in this study, to explore the neuroprotective mechanisms of HIF- 1α, we measured the expression level of these oxidant indicators by western blot. We used western blot to assess the expression level of these oxidant markers in the present study to investigate the neuroprotective mechanisms of HIF- 1α. The findings demonstrated that cerebral ischemia induced oxidative damage and weakened the antioxidant defense system, but KG diet supplementation prevented these effects following ischemic stroke. Additionally, it has been suggested that the complex TXNIP can sense an increase in ROS during ischemia and cause this complex to dissociate [59]. TXNIP is normally maintained in the reduced state in normal cells. When ROS generation is elevated, TXNIP dissociates from this complex and binds to the LRR region of NLRP3, activating the NLRP3 inflammasome [60]. The current study found that pre-treatment with the KG diet decreased the production of ROS following stroke and could lower the activation of the TXNIP/NLRP3 inflammasome, suggesting that the KG diet may protect brain damage from ROS-mediated NLRP3 activation. Thus, the neuroprotective effects of the KG diet may be attributed to the stabilization of HIF- 1α or/and activation of pro-survival pathways and inhibition of oxidative stress in the early stage of stroke.

The present work acknowledged that inflammation was not specifically compared between untreated WT and NLRP3 KO mice under MCAO settings. Our work primarily compared WT and NLRP3 KO mice under KG and MCAO conditions. However, earlier research has shown that in experimental stroke models, NLRP3 genetic deletion can lessen neuroinflammation and ischemic injury [17, 19, 61]. Our results support these observations since NLRP3 KO mice showed a tendency towards better behavioral outcomes and smaller infarcts. Future research should directly compare untreated WT and NLRP3 KO mice to completely clarify the role of the NLRP3 deficit in stroke prevention. The present study recognizes that this might affect how KG’s benefits are interpreted.

Alternative neuroprotective mechanisms beyond NLRP3 suppression may be involved, as evidenced by the non-significant decrease in infarct size observed in KG-treated NLRP3 KO mice. Although NLRP3 plays a significant role in post-stroke inflammation, there may be some residual protection even in the absence of NLRP3 due to compensatory pathways such as IL- 10/Jak-Stat stabilization and other inflammasomes like AIM2 and NLRC4 [54, 62]. Furthermore, KG-mediated metabolic modifications, like apoptosis and oxidative stress reductions through HIF- 1α stabilization, may endure without NLRP3. Future research could investigate whether KG still offers neuroprotection in NLRP3/AIM2 double knockout models and investigate the activation of alternative inflammasomes in NLRP3 KO mice. Furthermore, whether KG benefits go beyond NLRP3 regulation may become clearer with a more thorough examination of IL- 10-mediated anti-inflammatory responses in NLRP3 KO animals.

## Conclusion

**Fig. 10:**
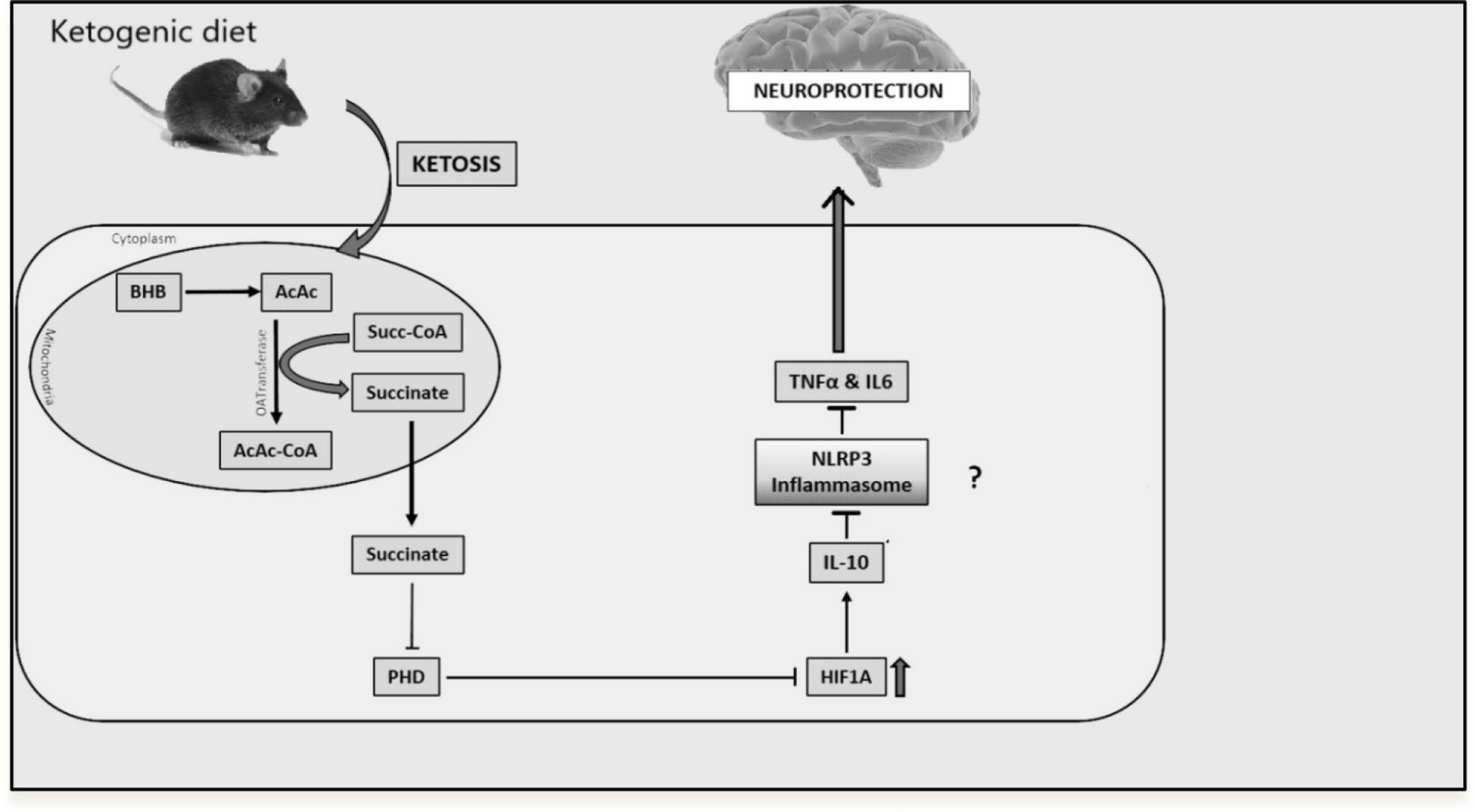
The molecular mechanisms behind the observed downregulation of IL- 6 and TNF-α by IL- 10 are highlighted in a schematic of our model system. NLRP3 is shown to be downregulated by HIF- 1α and IL- 10, which can support neuroprotection. The mechanism for HIF- 1α stabilization by the KG diet is through cellular metabolic redox signaling within the mitochondria. Particularly, succinate is a product of the catabolic process for ketone bodies that are produced at the citric acid cycle level after the activation of AcAc to AcAc-CoA as linked with the conversion of succinyl-CoA to succinate by CoA transferase (OAT). Succinate is moved from the mitochondria into the cytosol to maintain metabolic redox, inhibiting prolyl hydroxylases (PHD) and causing an accumulation of HIF- 1α. Ultimately, the molecular pathway involved in the HIF- 1α-mediated downregulation of pro-inflammatory cytokines –IL- 6 and TNF-α, is via IL- 10 mediated attenuation of NLRP3 inflammasome–mediated transcriptional attenuation

The results of this study show that IL- 10 overexpression was linked to HIF- 1α stabilization in KG-pre-treated animals. The KG diet could also prevent cerebral ischemic injury by reducing apoptosis, oxidative stress, and the neuroinflammatory response and enhancing behavioral outcomes. Although our results indicated that these protective benefits are probably due to the suppression of TXNIP/NLRP3 response via IL- 10, mediated by HIF- 1α, we also recognize the potential impact of baseline protection in NLRP3 KO mice and the potential for other protective mechanisms (Fig. 10). More investigation will be needed to break down these systems and ascertain whether KG still has neuroprotective effects in NLRP3-independent pathways. Moreover, KG treatment deactivates NLRP3 inflammasomes, which could be used as a novel therapeutic target for the clinical management of ischemic stroke. However, there is still insufficient information about the specific molecular mechanism of the KG diet. The underlying mechanisms must be further revealed before the KG diet can be used in clinical settings.

## Supplementary Information

Below is the link to the electronic supplementary material.

Supplementary file1 (DOCX 900 KB)

## Data Availability

Data will be made available upon reasonable request. The data used to support this study's findings are included in the article and available as supplementary material.

## Declarations

The institutional animal committee at UTHSC approved all animal studies procedures by the ethical guidelines of the National Institutes of Health for the care and use of laboratory animals.
