# Supplementary material for: Chronic Ketosis Provides Neuroprotection Through HIF- 1α-Mediated Control of the TXNIP/NLRP3 Axis by Regulating the Inflammatory and Apoptotic Response
Source: Mol Neurobiol. 2025 Apr 24;62(9):11238–52. doi: 10.1007/s12035-025-04943-0 (PMC12367822; doi:10.1007/s12035-025-04943-0)
Supplement: Supplementary file 1 — Supplementary file1 (DOCX 900 KB) [file 12035_2025_4943_MOESM1_ESM.docx]

**Table S1.** **The description of different parameters that were analyzed in the present study using the Catwalk XT system.**

| **Parameters** | **Description** |
| --- | --- |
| Run_ Average_ Speed | Speed of the animal’s body in the recorded run |
| Body Speed | Speed is calculated by dividing the distance that  the animal’s body traveled from one initial  contact of one paw to the next by the time to  travel that distance |
| Mean Intensity | The mean intensity of the complete paw |
| Coupling_ LF -> RH | The temporal relationship between the placements  of two paws within a step cycle. |

**Table S2: Effect of KG diet on body weight in experimental groups.**

| **Groups** | **Body weight (g)** | | | |
| --- | --- | --- | --- | --- |
|  | **0 h** | **24 h** | **48 h** | **72 h** |
| Sham | 26.54±0.46 | 27.36±0.51 | 27.66±0.57 | 27.34±0.54 |
| pMCAO | 28.12±0.47  (+5.97%) | 27.35±0.52  (-1.41%) | 27.51±0.56  (-2.16%) | 27.34±0.61  (-1.15%) |
| KG+pMCAO | 30.87±0.47  (+9.76%) | 29.88±0.54  (+11.37%) | 29.87±0.61  (+10.43%) | 29.87±0.61  (+10.53%) |
| KG | 30.94±0.48 | 30.96±0.56 | 31.02±0.60 | 31.04±0.51 |

The body weight changes were recorded in all experimental groups at different time intervals before, 24 hours, 48 hours, and 72 hours after stroke. Values are expressed as mean ± SEM (n=6-9).

**Figure-1S**

**
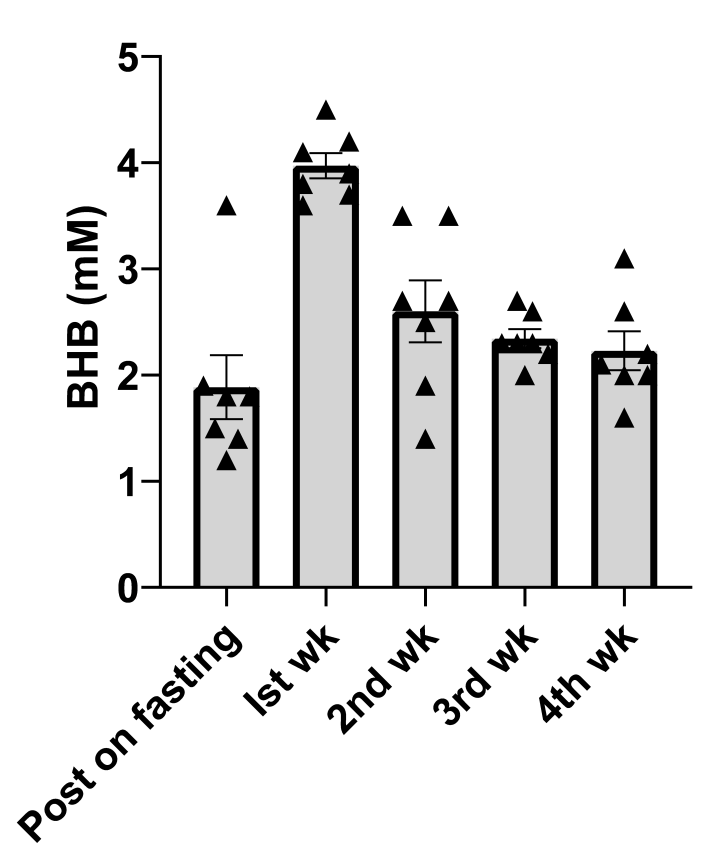
A.**

**B.**

**
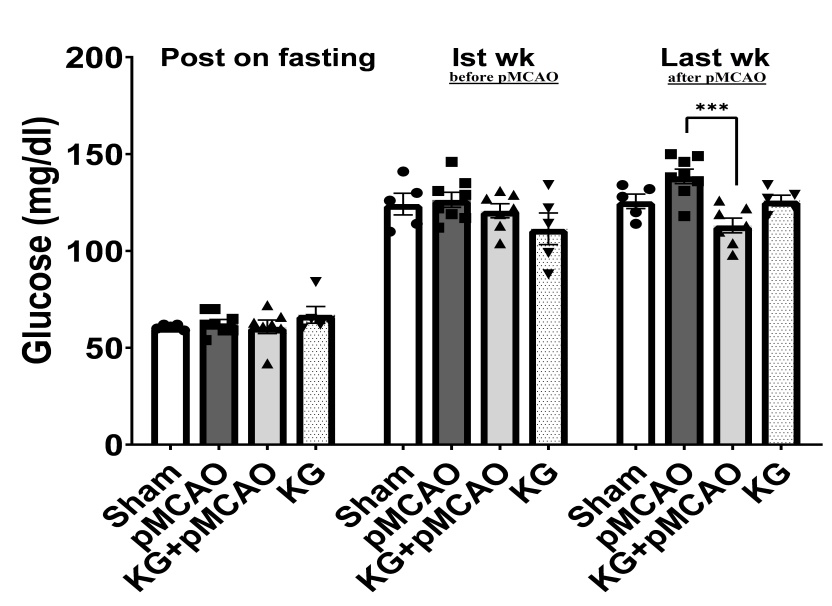
**

**Figure 1S. A.** Blood ketones (BHB) in mice fed with the KG diet were measured with Precision Xtra® hand-held keto-monitor. The blood ketone levels in KG-fed mice spiked in the first week and stabilized in the following weeks. **B.** Blood glucose levels were measured using Precision Xtra® handheld glucometer. Results showed that glucose levels were elevated in the pMCAO compared to the KG+pMCAO mice. The values were presented as the mean ± S.E.M. (n=8-9). *** Denotes statistical significance (p<0.001).

**Figure 2S.**

**
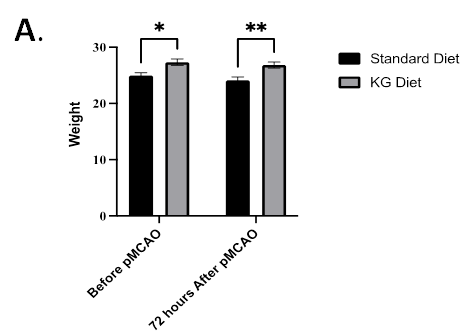

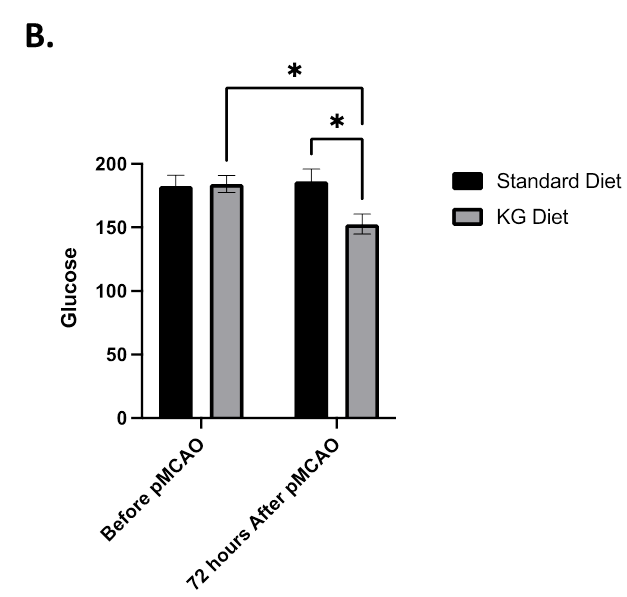
**

**
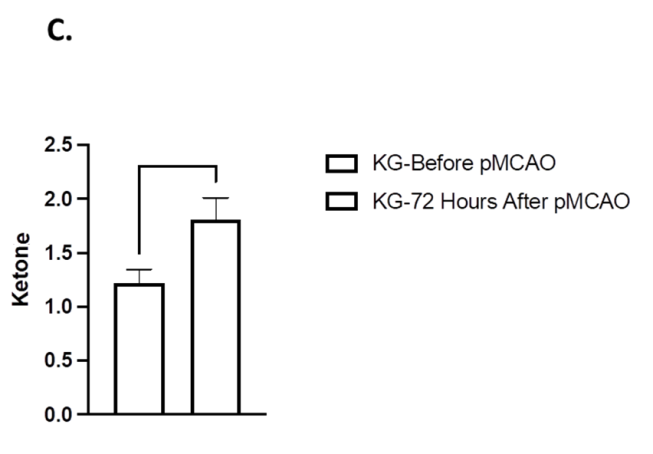
**

**Figure 2S: Effect of genetic manipulation of NLRP3 on the metabolic panel following PMCAO.** NLRP3 knock-out mice showed significant restoration of body weight in KG group animals (Figure 2S A). After KG supplementation, body weight was significantly reduced following pMCAO at 72 hrs. We also observed significant changes in body weight before pMCAO in KG-supplemented animals compared to the STD group. Interestingly, the KG diet did not change fasting blood glucose levels (data not shown) before PMCAO in KG group animals. At the same time, blood glucose concentration was significantly lower in the KG group compared to the STD group after 72 hrs (Figure 2S B). Blood ketone level was found to be non-significant in KG group mice compared to STD after 72 hrs (Figure 2S C).

**Figure 3S.**


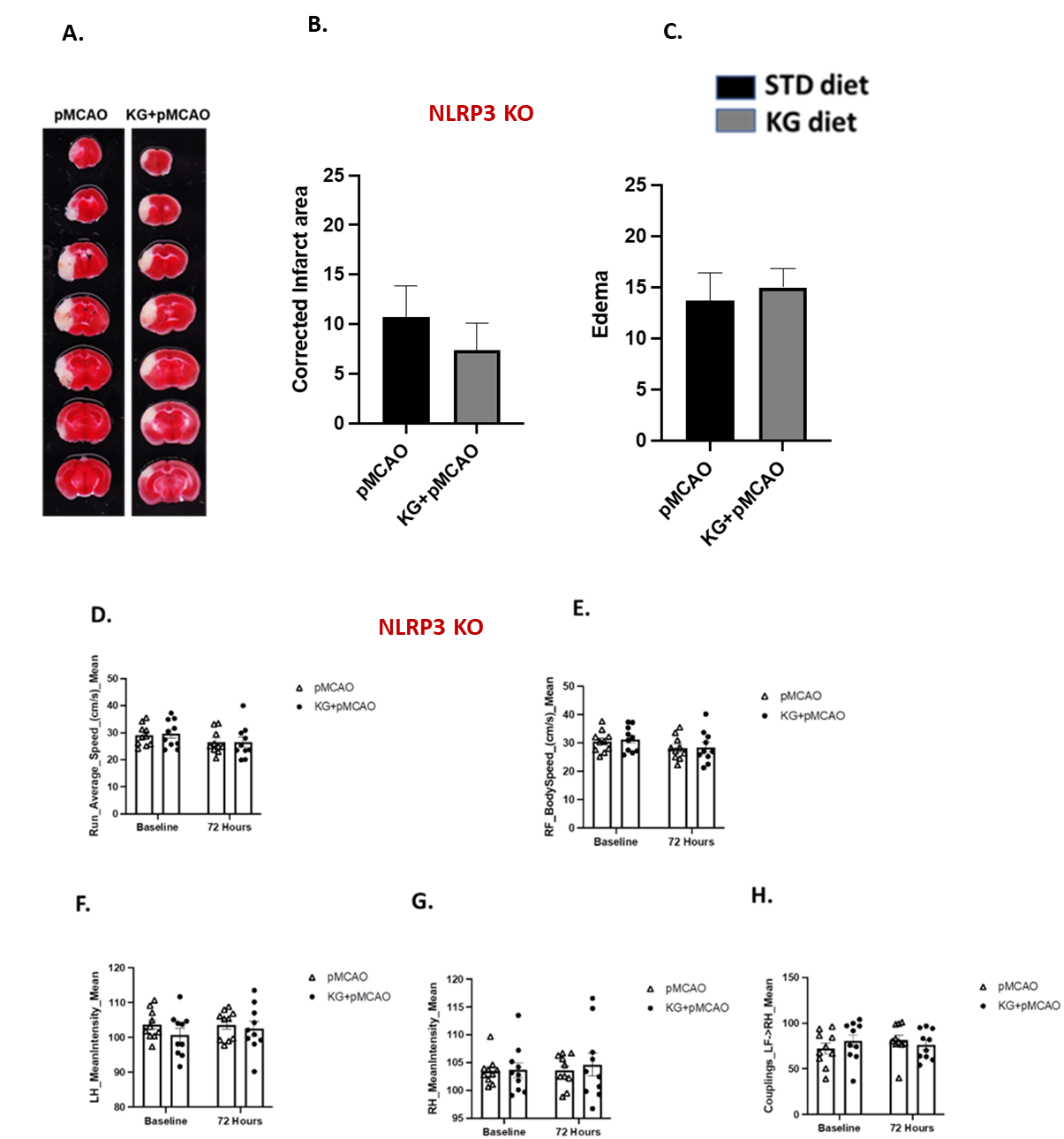


**WT vs NLRP3 KO mice**

**
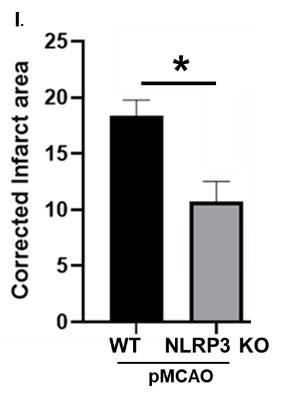
**

**Figure 3S: KG-diet mediates partial neuroprotective effects through NLRP3 inflammasome.** KG-diet showed non-significant trends in infarction, edema, and better sensory-motor performance in NLRP3 knockout mice at 72 hrs after photothrombotic stroke. **(A).** TTC sections of brain samples in pMCAO and KG+pMCAO group. **(B).** KG+pMCAO showed a remarkable reduction in infarct size compared to the pMCAO. **(C).** KG+pMCAO represented a decrease in ipsilateral edema post-injury. (**D, E, F, G, & H)** Our data represented non-significant sensory-motor performance changes measured through different parameters in either the pMCAO or KG+ pMCAO group. (**I**) NLRP3 KO mice itself showed a decrease in infarct area as compared to WT mice at 72h after pMCAO. Values are expressed as mean ± SEM (n=10**).**

**Full uncropped Gels and Blots image**


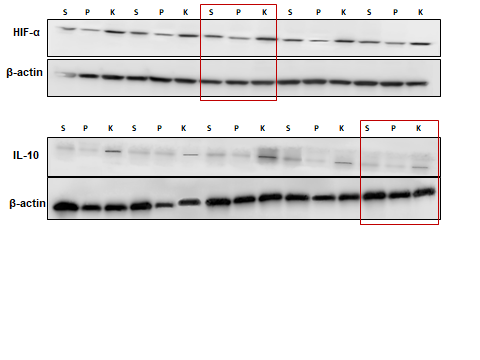


**Figure 4S**. Effect of KG diet in pMCAO mice. The KG diet modulated HIF-1α, which was associated with high expression of IL-10 in the KG+pMCAO group. The relative intensity of the targeted protein bands was normalized to the β-actin. HIF-1α, Hypoxia-Inducible Factor-1 alpha; interleukin-10, IL-10. S, sham; P, STD diet + photothrombotic stroke (pMCAO group); K, KG diet + photothrombotic stroke (KG+pMCAO). (n=5).


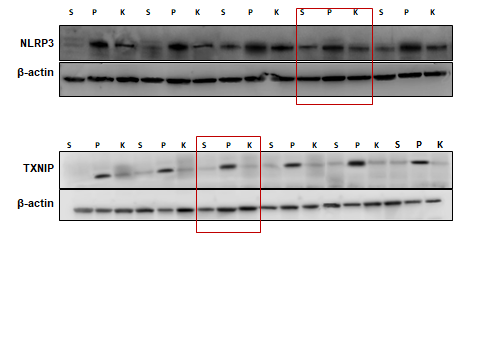


**Figure 5S**. KG diet downregulated the expression of NLRP3/TXNIP axis in pMCAO mice. The relative intensity of the targeted protein bands was normalized to the β-actin. NLRP3, NOD-like receptor pyrin domain-containing-3; TXNIP, Thioredoxin-interacting protein. S, sham; P, STD diet + photothrombotic stroke (pMCAO group); K, KG diet + photothrombotic stroke (KG+pMCAO). (n= 5-6)


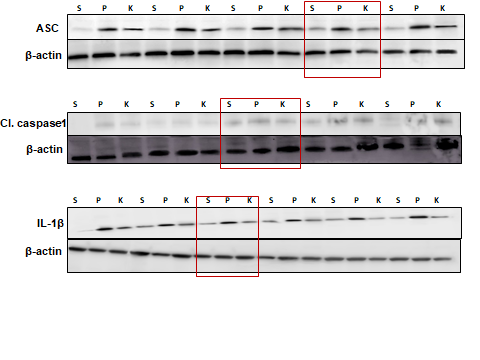


**Figure 6S.** KG diet modulated NLRP3 inflammasome-associated proteins in pMCAO mice. The relative intensity of the targeted protein bands was normalized to the β-actin. ASC, apoptosis-associated speck-like protein; Cl-caspase-1, cleaved caspase-1; IL-1β, interleukin-1β. The relative intensity of the targeted protein bands was normalized to the β-actin. S, sham; P, STD diet + photothrombotic stroke (pMCAO group); K, KG diet + photothrombotic stroke (KG+pMCAO). (n= 5-6).


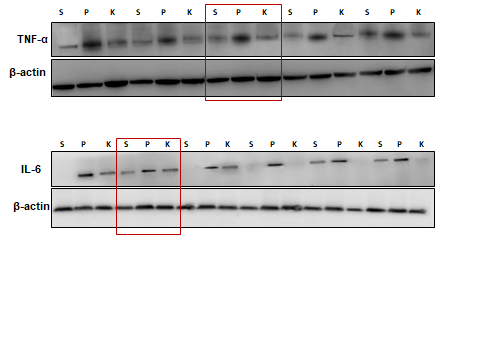


**Figure 7S.** KG diet preconditioning significantly downregulated the pro-inflammatory cytokines in pMCAO mice. The relative intensity of the targeted protein bands was normalized to the β-actin. TNF-α, tumor necrosis factor alpha; IL-6, interleukin-6. S, sham; P, STD diet + photothrombotic stroke (pMCAO group); K, KG diet + photothrombotic stroke (KG+pMCAO). (n= 5-6).


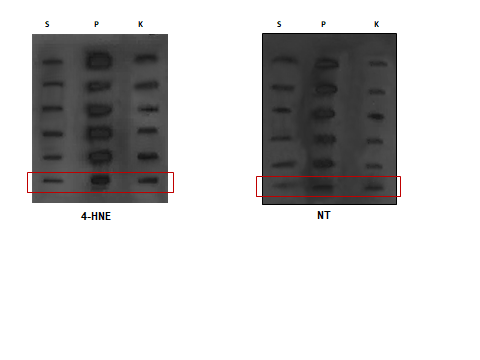


**Figure 8S.** KG diet attenuates oxidative stress in pMCAO mice. (A) Representative immunoblot indicates the relative intensity of 4-HNE and NT expression levels in the penumbra brain tissue lysate. The relative intensity of the targeted protein bands was normalized to ponceau staining. NT, Nitrotyrosine; 4-HNE, 4-hydroxynonenal. S, sham; P, STD diet + photothrombotic stroke (pMCAO group); K, KG diet + photothrombotic stroke (KG+pMCAO). (n= 6).
